# Refined expression quantitative trait locus analysis on adenocarcinoma at the gastroesophageal junction reveals susceptibility and prognostic markers

**DOI:** 10.3389/fgene.2023.1180500

**Published:** 2023-05-17

**Authors:** Ce Zhong, Chen Wu, Yuan Lin, Dongxin Lin

**Affiliations:** ^1^ Department of Etiology and Carcinogenesis, National Cancer Center/National Clinical Research Center for Cancer/Cancer Hospital, Chinese Academy of Medical Sciences and Peking Union Medical College, Beijing, China; ^2^ Beijing Advanced Innovation Center for Genomics (ICG), Biomedical Pioneering Innovation Center (BIOPIC), Peking University, Beijing, China

**Keywords:** adenocarcinoma at the gastroesophageal junction, cell deconvolution, cell type–specific expression quantitative trait loci, linear mixed model, susceptibility biomarkers, prognostic prediction

## Abstract

**Objectives:** This study aimed to explore cell type level expression quantitative trait loci (eQTL) in adenocarcinoma at the gastroesophageal junction (ACGEJ) and identify susceptibility and prognosis markers.

**Methods:** Whole-genome sequencing (WGS) was performed on 120 paired samples from Chinese ACGEJ patients. Germline mutations were detected by GATK tools. RNA sequencing (RNA-seq) data on ACGEJ samples were taken from our previous studies. Public single-cell RNA sequencing (scRNA-seq) data were used to produce the proportion of epithelial cells. Matrix eQTL and a linear mixed model were used to identify condition-specific cis-eQTLs. The R package coloc was used to perform co-localization analysis with the public data of genome-wide association studies (GWASs). Log-rank and Cox regression tests were used to identify survival-associated eQTL and genes. Functions of candidate risk loci were explored by experimental validation.

**Results:** Refined eQTL analyses of paired ACGEJ samples were performed and 2,036 potential ACGEJ-specific eQTLs with East Asian specificity were identified in total. ACGEJ-gain eQTLs were enriched at promoter regions more than ACGEJ-loss eQTLs. rs658524 was identified as the top eQTL close to the transcription start site of its paired gene (*CTSW*). rs2240191–*RASAL1*, rs4236599–*FOXP2*, rs4947311–*PSORS1C1*, rs13134812–*LOC391674*, and rs17508585–*CDK13-DT* were identified as ACGEJ-specific susceptibility eQTLs. rs309483–*LINC01355* was associated with the overall survival of ACGEJ patients. We explored functions of candidate eQTLs such as rs658524, rs309483, rs2240191, and rs4947311 by experimental validation.

**Conclusion:** This study provides new risk loci for ACGEJ susceptibility and effective disease prognosis biomarkers.

## Introduction

Many SNPs (single-nucleotide polymorphisms) have been linked to complex diseases such as diabetes, Alzheimer disease, and multiple cancers (Wright et al., 2019; Forrest et al., 2022; Wang et al., 2022). GWASs reveal hundreds of risk loci, most of which are located in non-coding regions of the DNA, suggesting that deciphering their biological functions remains challenging (Ong et al., 2022; Sollis et al., 2023). eQTL is a commonly used method to explain potential functions of risk loci identified by GWASs (Umans et al., 2021). This approach is based on the concept that a GWAS variant in some tissues affects expression at a nearby gene and that both the gene and tissue might play a role in the disease mechanism (Gamazon et al., 2015; Ratnapriya et al., 2019). However, previous studies have mostly investigated eQTLs at the tissue level, leading to ever growing sample sizes but partially successful in prioritizing disease risk SNPs and genes (Human genomics, 2015; Battle et al., 2017). Recent studies have shifted focus on cell type–specific eQTLs because many disease-associated eQTLs have been observed in specific cell types, and these eQTLs show strong enrichment for heritability across complex traits (Hormozdiari et al., 2018; Sarkar et al., 2019; Cuomo et al., 2020; van der Wijst et al., 2020; Neavin et al., 2021; Ota et al., 2021; Bryois et al., 2022; Yazar et al., 2022). Moreover, despite non-coding RNAs, especially long non-coding RNAs (lncRNAs), being regarded as tissue-specific prognosis markers and therapeutic targets of many kinds of tumors, eQTL analyses focusing on non-coding genes still remain exiguous (de Goede et al., 2021; Statello et al., 2021).

ACGEJ has had a rapidly increasing incidence in western countries during the past few decades (Chevallay et al., 2018). In Asia, where squamous cell carcinoma is the predominant type of esophageal cancer, the rising incidence of ACGEJ has also been reported (Rashkin et al., 2020; Sakaue et al., 2021). The five-year survival rates of this cancer are 20–25%, which is lower than that of esophageal or gastric cancers (Lin et al., 2020). Previous tissue level studies have identified susceptibility loci and therapeutic genes for ACGEJ (Abnet et al., 2010; Wang et al., 2010a; Hu et al., 2016; Lott and Carvajal-Carmona, 2018; Lin et al., 2020; Ku et al., 2021; Lao et al., 2022). However, most eQTL studies of tumors lack enough resolution and paired adjacent normal samples (Gong et al., 2018; Calabrese et al., 2020). Therefore, deciphering tumor-specific eQTLs may help elucidate carcinogenic mechanisms and inform broadly applicable risk assessment efforts.

In the present study, we conduct an eQTL study on ACGEJ using WGS, RNA-seq, and public scRNA-seq data. To increase the resolution of eQTLs at the cell type level, we use a proportion of the epithelial cells multiplying genotypes as an interaction term. Epithelial cell proportions are generated by computational deconvolution using reference gene expression profiles produced by scRNA-seq data. To increase the resolution of eQTLs at the disease level, we estimate effects of epithelial cell–specific eQTLs on ACGEJ and normal cells. Here, we reveal substantial ACGEJ-specific effects (ACGEJ-loss/ACGEJ-gain eQTLs) in the genetic regulation of gene expression. The integration of cell type–specific eQTLs with GWAS and clinical data identifies susceptibility and prognosis markers of ACGEJ. The present study provides insights into tumor-specific eQTLs and identifies risk loci more precisely and specifically than tissue level studies.

## Materials and methods

### Biospecimens and clinical data

The biospecimens used in this study were collected from 120 Chinese ACGEJ patients at the Linzhou Cancer Hospital and Linzhou Esophageal Cancer Hospital (Henan province, China). Tumor, adjacent normal tissue, and peripheral blood samples were obtained by surgical resection. ACGEJ was confirmed by at least two pathologists via histopathological examination. Patients received no chemotherapy or radiotherapy before surgery. Clinical data were collected from the medical record of each patient (Supplementary Table S1).

### DNA and RNA sequencing data

Aligned DNA sequences (as bam files) and gene expression data were obtained from a previous study of our group (Lin et al., 2020). In summary, blood DNA was extracted using the QIAamp DNA Blood Midi Kit (QIAGEN), and total RNA was extracted from tissue samples using the AllPrep DNA/RNA Kit (QIAGEN). Whole-genome library preparation was carried out using the TruSeq Nano DNA HT Sample Preparation Kit and 500 ng of genomic DNA per sample sheared into 350-bp fragments. The RNA library preparation was carried out using the NEBNext^®^ Ultra™ Directional RNA Library Prep Kit for Illumina and 3 ng of RNA per sample as the initial material. Ribosomal RNA was removed using the Epicenter Ribo-Zero™ Gold Kits. Library fragments were purified using the kaiao K5500^®^ Spectrophotometer (Kaiao, Beijing, China). All libraries were sequenced on an Illumina Hiseq 4000 platform, and 150-bp paired-end reads were generated.

### Differential gene expression analysis

We considered a gene as differentially expressed in tumor and normal samples if the log2 + 1 transformed TPM (transcript per million) change was significant in the Wilcoxon test with the adjusted *q*-value (FDR, false discovery rate) was <0.05 and the fold change of mean TPM was >1.2 or <0.8.

### SNP calling

Germline mutations were detected using GATK tools (v4.2.0.0) (Poplin et al., 2018). From biallelic SNPs that passed variant quality score recalibration, we used PLINK (v1.9) (Purcell et al., 2007) to remove those with minor allele frequency <0.01 or missing call rate >5% or those deviating from the Hardy–Weinberg equilibrium with a *p*-value <10^−6^. We further removed SNPs in the Y chromosome and SNPs with the minor allele observed in <10 patients. We referred to the GENCODE v23 basic gene annotation for genomic coordinates of the transcription start site (TSS) and end site (TES) and converted the SNP coordinates from hg19 to hg38 using the liftOver function of the R rtracklayer (version 1.46.0) package (Hinrichs et al., 2006).

### Cell type deconvolution, epithelial cell proportion, and high-resolution gene expression

To de-convolve cluster-specific cell subsets from bulk RNA-seq, signature matrices of epithelial, immune, and stromal cells were calculated using the public scRNA-seq data by CIBERSORTx (Newman et al., 2019). A total of 3,665 epithelial, 321 immune, and 53 stromal cells were taken from three clusters using the Seurat (v4.2.0) (Hao et al., 2021) subset function and labeled with the corresponding cluster identities. Cluster-labeled cells were used to obtain a single-cell reference matrix that was used as an input and run on the CIBERSORTx online server using the ‘Custom’ option. The disable quantile normalization option was used. The default value for sampling (0.5) was used. Replicates (Ratnapriya et al., 2019) and minimum expression (0) were used. Additional options for kappa (999), q-value (0.01), and the number of barcode genes (300–500) were kept at default values. We obtained consistent results on cell proportion using some other references and parameter settings (Supplementary Figures S1A,B) (Sathe et al., 2020). The CIBERSORTx single-cell reference matrix was used to impute cell fractions from the bulk RNA-seq of mixture files. The mixture files (TPM values) were used as the input and run on the CIBERSORTx online server using the “Impute Cell Fraction” analysis with the “Custom” option selected, and the S-mode batch-correction was applied. Cell fractions were run in the relative mode to normalize results to 100%. The number of permutations to test for significance was kept at default (100). The estimated epithelial cell fraction is shown in Supplementary Table S8 and is highly correlated with that inferred using the ESTIMATE (Supplementary Figure S2A) (Yoshihara et al., 2013). Mixture files, signature matrices, and cell fractions were used to obtain cell expressions using the “Impute Cell Expression” analysis with the “Custom” and “High-Resolution” options selected. A list of genes was used as the ‘gene subset file,’ and the S-mode batch-correction was applied. Due to the limitation of CIBERSORTx on low-expressed (mRNA level of most samples <1) genes, we deleted those genes and transcription factors where the mRNA levels of most samples was <1 before the differential gene expression analysis.

### Genome-wide identification of epithelial cell fraction–dependent cis-eQTLs

We used the Matrix eQTL (v2.3) to identify epithelial cell–specific and ACGEJ-specific eQTL. Patients’ age (grouped as < or ≥ median), sex, drinking status, smoking status, and estimated epithelial cell fraction were included as covariates. Therefore the first two genotype PCs in addition to the first 30 PEER factors for ACGEJ samples and the first three genotype PCs in addition to the first 10 PEER factors for normal tissue samples were applied to capture the hidden technical variations and resultant batch effects. PCs and PEER factors were inferred using the snpgdsPCA function in the R SNPRelate package (v1.20.1) (Zheng et al., 2012) and the R peer package (v1.0) (Stegle et al., 2010), respectively. We applied the LINEAR_CROSS model of Matrix eQTL to test for the significance of the interaction between the genotype at a specific locus and the estimated epithelial cell fraction (as the last covariate in order), so as to identify cis-eQTLs whose effects depend on the enrichment of the epithelial cells. Consistent with an earlier study on this matter (Geeleher et al., 2018), this model effectively controlled *p*-value inflation (Supplementary Figures S3A,B) and thus the false-positive rate. Since we used all SNPs extracted from the WGS data without conducting LD pruning, a hierarchical multi-test correction procedure (Huang et al., 2020) was then adopted to adjust the nominal *p*-values from the Matrix eQTL, and the SNPs with the adjusted *p* ≤ 0.01 were considered cis-eQTLs. We performed the GSEA enrichment analysis of epithelial cell–specific eQTLs, which revealed the epithelial cell specificity of our result (Supplementary Figures S3C). Parameters such as the minimum gene set size (minGSSize), maximum gene set size (maxGSSize), permutation (nPerm), and *p*-value cut-offs were the default values. The *p*-value adjustment method was set to Benjamini and Hochberg (BH).

### ACGEJ-associated cis-eQTL identification

We looked for significant associations between eQTL genotype and gene expression conditional on the disease status (ACGEJ/normal) by combining significant ePairs separately identified in the ACGEJ and normal samples into an LMM and then testing the significance of an interaction term between the eQTL genotype and disease status. To decrease probable redundancy due to LD, we did not include all the earlier identified cis-eQTLs but only the most significant one (top eQTL) for each gene in either disease status. As for genome-wide cis-eQTL identification, we used linear models to account for the 17 covariates common in the earlier ACGEJ/normal sample–exclusive cis-eQTL mapping, such as age, sex, drinking and smoking status, estimated epithelial cell fraction, the first two genotype PCs, and the first 10 PEER factors. However, now that the samples had been paired and were therefore non-independent, we built LMMs using the lmer function from the lme4 R package (v1.1-30) (Huang et al., 2020). Specifically, we fit two models as given below—one with and the other without the interaction between the genotype and disease status (ACGEJ = 1 and normal = 0):

$$expression\;∼\;genotype+status+genotype\times status+\sum_{k=1}^{17}\left({covariate}_{k}+status\times {covariate}_{k}\right)+\left(1|Sample\right)$$

This model allows detecting eQTLs with different effects between two disease statuses, while avoiding the trouble of untangling every possible disease-associated confounder. We applied a permutation-based method (Huang et al., 2020) to estimate the empirical *p*-values for the genotype–status interaction and the required *p-*value < 0.05 for statistical significance.

### Annotation of SNPs and genes

Germline SNPs were annotated by ANNOVAR (24 October 2019) (Wang et al., 2010b). Genes were annotated by BioMart Ensembl gene 108 (Durinck et al., 2009).

### Cancer-related gene sets

The Hallmark gene set was downloaded from Human MSigDB Collections (v2023.1.Hs) (Subramanian et al., 2005; Liberzon et al., 2015). The version of the COSMIC gene set was v96, released on 31 May 2022 (Tate et al., 2019). The cancer susceptibility gene set was downloaded from the CSGs v1.0 in March 2022 (Shi et al., 2022). The therapeutic gene set was downloaded from OncoKB (v3.0) (Chakravarty et al., 2017) (Supplementary Table S2).

### Gene set variation analysis

The gene set variation analysis was performed by using the R package GSVA (v1.42.0) (Hänzelmann et al., 2013) and limma (v3.50.3) (Ritchie et al., 2015).

### GWAS co-localization

GWAS co-localization was performed by the R package coloc (v5.1.0.1) (Wallace, 2021). The GWAS data sets were downloaded from published GWAS data sets such as GCST90018841 (Sakaue et al., 2021), GCST90018848 (Sakaue et al., 2021), GCST90018849 (Sakaue et al., 2021), GCST90000514 (Ong et al., 2022), and GCST90000515 (Ong et al., 2022) and the CCGD-ESCC (Peng et al., 2018) and public databases such as bbj-a-117 (Nakamura, 2007). The traits of GWAS that we used included esophageal cancer, gastroesophageal reflux disease, Barrett’s esophagus, and gastric carcinoma (Supplementary Table S6).

### Bayesian fine-mapping

We performed fine-mapping based on summary statistics and matched the LD matrix for the top SNP rs658524 and SNPs with high LD to rs658524 (LD > 0.8) using Fine-mapping (Huang et al., 2017). We used the expression of *CTSW* to be the trait and adjusted this model with the age of the sample, obtaining sex, smoking status, drinking status, TNM stage, and epithelial cell proportion. We used the recommended parameters of the tool. Fine-mapping reports the PIP (posterior inclusion probability) of each variant that is causal in the specific model. We recorded potentially causal variants in a 99% credible set according to the fine-mapping PIP (Supplementary Table S9).

### Public scRNA-seq data

The scRNA-seq data were downloaded from the published article of Nowicki-Osuch et al. (2021). The cells were collected from the normal gastric cardia data set. We used the clustering result of the original study. We combined 3,665 glandular epithelial cells, 321 immune cells, and 53 stromal cells as the epithelial, immune, and stromal cell cluster, respectively.

### Cell lines and cell culture

The human ACGEJ cell line OE19 was purchased from the Cell Resource Center, IBMS, CAMS/PUMC, Beijing, China. The cells were cultured in Dulbecco’s modified Eagle’s medium (DMEM) supplemented with 10% fetal bovine serum.

### *CTSW* genotyping

The *CTSW* rs658524G/A genotypes were identified by PCR with the primers shown in Supplementary Table S3.

### Small interfering RNA transfection

Small interfering RNA (Supplementary Table S3) targeting *KLF5* was obtained from GenePharma. The transfection of siRNAs was performed using the jetPRIME transfection reagent (#101000046, Polyplus, France).

### Real-time quantitative PCR

Total RNA extraction and reverse transcription were performed using the RNA-Quick Purification Kit (RN001, ES Science) and PrimeScript RT reagent Kit (RR036A, TaKaRa), respectively. Quantitative PCR (qPCR) was performed in triplicate using TB Green Premix Ex Taq II (RR820A, TaKaRa) with the primers shown in Supplementary Table S3.

### Western blot analysis

Total protein was subjected to SDS-PAGE and transferred to the PVDF membrane (IPVH00010, Millipore). The antibody against CTSW (ab191083), KLF5 (21017-1-AP), or vinculin (ab219649) was obtained from Abcam or Proteintech. The membrane was incubated with the primary antibody and visualized using the Chemiluminescent Substrate Kit (34580, Thermo Fisher).

### Electrophoretic mobility-shift assays

Nuclear proteins were extracted from OE19 cells using the Nuclear and Cytoplasmic Protein Extraction Kit (P0028, Beyotime). Electrophoretic mobility-shift assays (EMSA) were performed using the Chemiluminescent EMSA Kit (#GS009, Beyotime). The probe sequences are shown in Supplementary Table S4.

### Construction of reporter plasmids and reporter assays

DNA fragments containing rs658524A or rs658524G (526 base pairs from the position −454 to +71) produced by DNA synthesis were cloned into the pGL3-Enhancer. DNA fragments containing rs4947311T, rs4947311C (600 base pairs from the position −299 to +300), rs2240191G, rs2240191T (600 base pairs from the position −299 to +300), rs309483G, or rs309483A (600 base pairs from the position −299 to +300) produced by DNA synthesis were cloned into the pGL3-Promoter. The luciferase reporter assays were performed according to the manufacturer’s instructions (Promega E1960).

### Chromatin immunoprecipitation–coupled qPCR analysis

Chromatin immunoprecipitation (ChIP) assays were performed using the SimpleChIP Plus Sonication Chromatin IP Kit (#56383, CST). OE19 cells were treated with formaldehyde for cross linking followed by ChIP with the KLF5 antibody or rabbit IgG. DNA fragments were analyzed by qPCR with the primers shown in Supplementary Table S3.

### Survival analysis

We followed up with 81 of the 120 ACGEJ patients. The overall survival time of these patients was estimated by using the Kaplan–Meier method, and the differences were examined by the log-rank test. The HR and 95% CI were calculated with the Cox proportional hazards model, adjusted by clinical data such as age, sex, smoking status, drinking status, and TNM stage (AJCC/UICC, STAD, v7).

### Statistical analysis

The unpaired *t*-test and Mann–Whitney test were used for the independence test between variables of the two unpaired gene expression groups. The Wilcoxon matched-pairs signed-rank test was used for the independence test between two paired gene expression groups (tumor and adjacent normal epithelial cell). All statistical tests were two-tailed unless specifically indicated, and a *p-*value < 0.05 was considered significant. All the statistical analyses were performed using the R-4.1.3 software.

### Other analyses

The LD r2 between two SNPs was calculated by using the snpgdsLDpair function in the R SNPRelate package. All statistical tests were two-tailed, and a *p*-value < 0.05 was considered significant, unless specifically indicated. All statistical analyses were conducted using the R (v 4.1.3) software. Potential transcription factors of *CTSW* that might bind to rs658524 were predicted by JASPAR 2022 (Castro-Mondragon et al., 2022) and AnimalTFDB (v4.0) (Shen et al., 2023) using the DNA fragment containing rs658524G (25 base pairs from the position −84 to −60).

## Results

### Identification of ACGEJ-specific eQTLs

**FIGURE 1:**
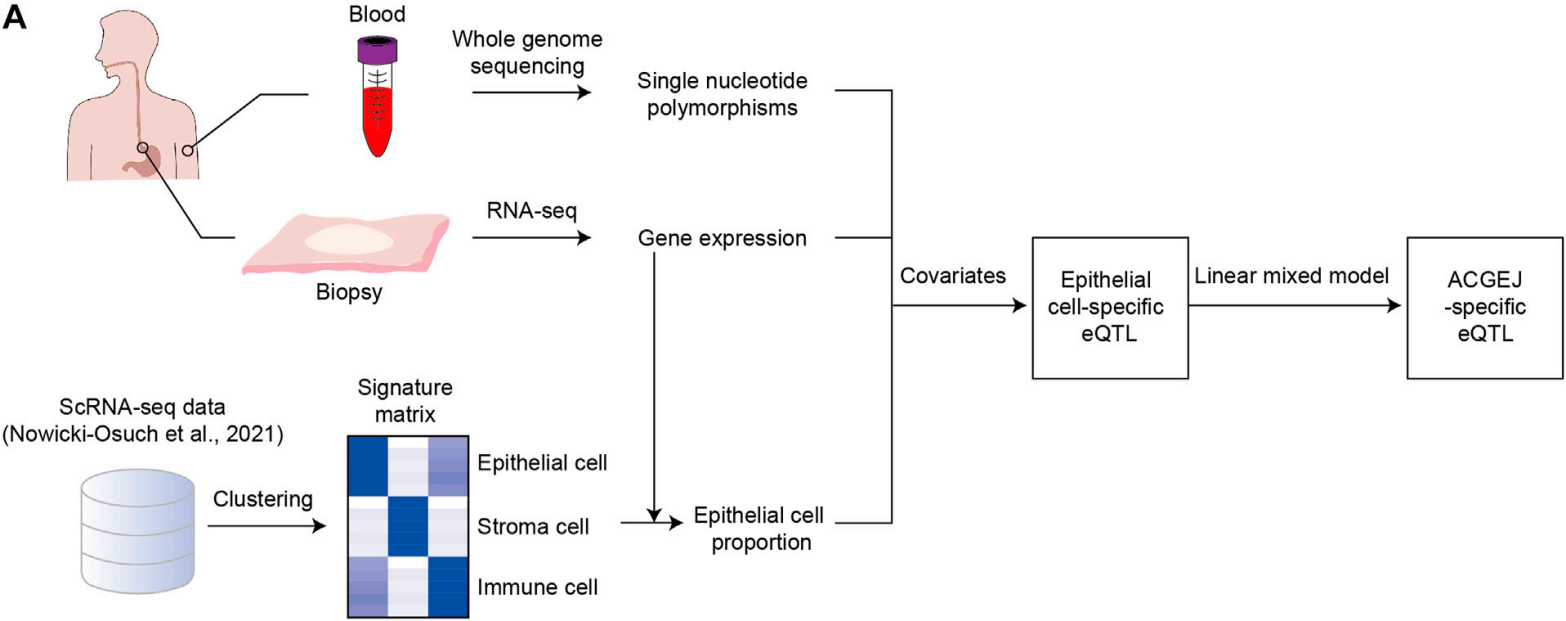
Identification of ACGEJ-loss/ACGEJ-gain eQTLs. **(A)** Flow chart of identification of ACGEJ-loss/ACGEJ-gain eQTLs. Peripheral blood samples were used to generate SNP data. Tumor and adjacent normal biopsy samples were used to generate gene expression data. Bulk gene expression and public scRNA-seq data from Nowicki-Osuch et al. (2021) were used to generate epithelial cell proportions. SNP, bulk gene expression, and epithelial cell proportion data were used to generate eQTLs together with covariates that included clinical data and PEER factors. A mixed linear model was used to generate ACGEJ-loss/-gain eQTLs.

**FIGURE 2:**
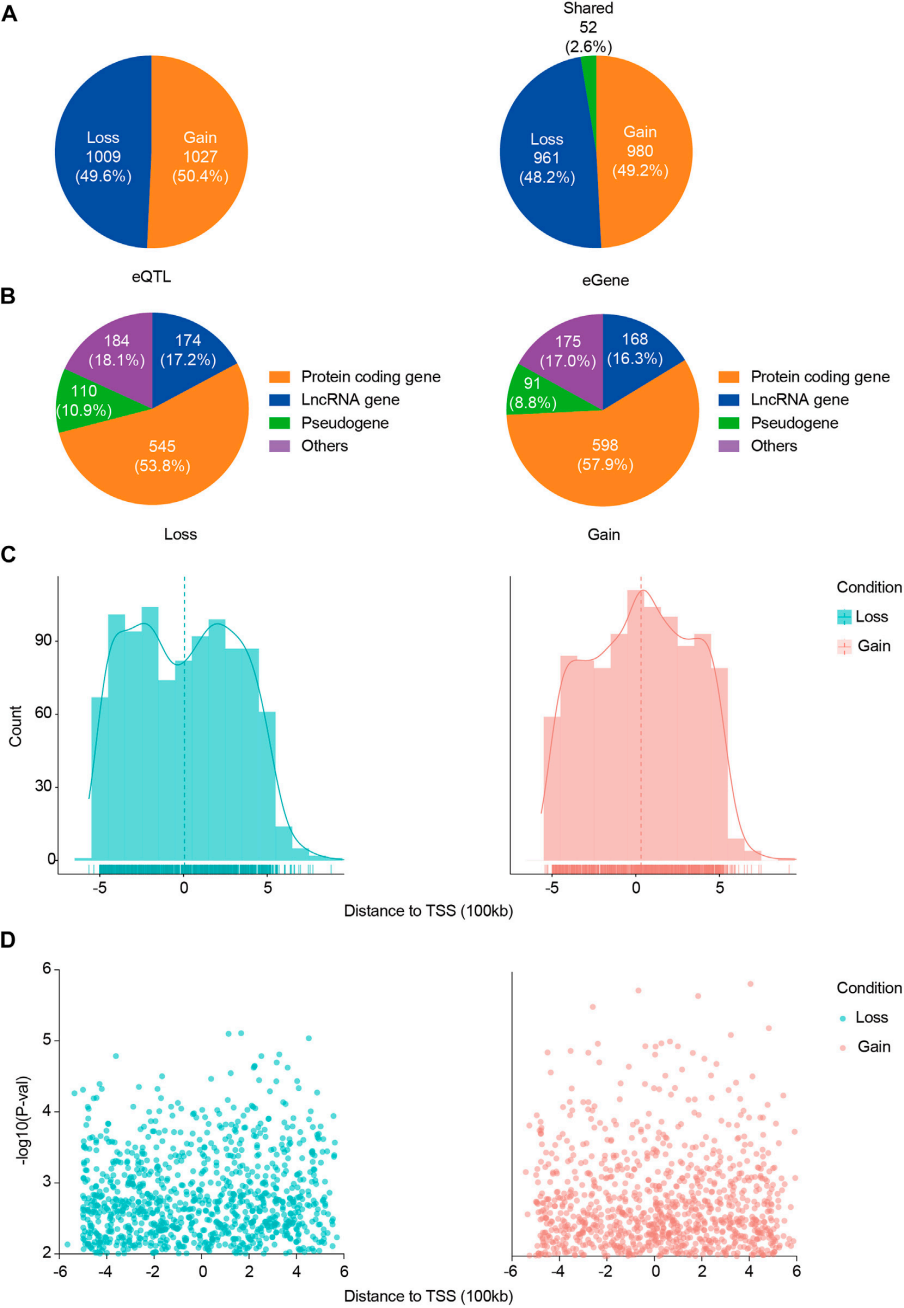
Distribution of ACGEJ-loss/ACGEJ-gain eQTLs and genes. **(A)** Exact numbers and proportions of ACGEJ-loss/ACGEJ-gain eQTLs (left) and genes (right). **(B)** Functional annotations of ACGEJ-loss (left) and ACGEJ-gain (right) genes. **(C)** Cell type specificity and distance of eQTL to TSS of their paired genes. *y*-axis: eQTL count; *x*-axis: distance from eQTLs to TSS of their paired genes. Density is highlighted by density regression curve. **(D)** Cell type specificity and distance of eQTL to TSS of their paired genes. *y*-axis: eQTL effect size [−log10 (*p*-value)] and *x*-axis: distance from eQTLs to TSS of their paired genes.

We first identified 125,164 eQTLs using the SNP set of 120 ACGEJ patients and the gene expression data of their ACGEJ samples and also identified 108,057 eQTLs using the same set of SNPs and the gene expression data of their normal tissue samples separately (Figure 1A). Then, we used a linear mixed model on the union of these ePairs and identified 2,045 ACGEJ-specific ePairs, involving 1,993 genes and 2,036 eQTLs (Supplementary Table S5). We further identified 1,009 (49.6%) ACGEJ-loss eQTLs and 1,027 (50.4%) ACGEJ-gain eQTLs (Figure 2A). ACGEJ-specific eQTLs consisted of 1,603 (78.7%) SNPs and 433 (21.3%) indels. We found that the top four eQTLs sorted by counts included A>G (16.4%), T>C (14.4%), C>T (11.0%), and G>A (11.0%) (Supplementary Table S5). Of all ACGEJ-specific eQTLs, we found 0.6% (12/2,036) were overlapped with the GTEx database and 0.2% (4/2,036) were novel variants (chr1: 144691189T>TA, chr1: 143770602AGG​TAT​ATC​TTG>A, chrX: 446457G>A, and chrX: 1251859G>GAA).

Meanwhile, we obtained 961 (48.2%) ACGEJ-loss genes and 980 (49.2%) ACGEJ-gain genes. Notably, 52 genes (2.6%) were found simultaneously in different ePairs under the ACGEJ or normal condition (Figure 2B). For further exploration of potential mechanisms of these genes, we annotated these genes and found that a fair number of genes were pseudogenes and non-coding RNAs. Of all ACGEJ-loss genes, 545 (53.8%), 174 (17.2%), and 110 (10.9%) were protein-coding genes, lncRNA genes, and pseudogenes, respectively (Figure 2B). Of all ACGEJ-gain genes, 598 (57.9%), 168 (16.3%), and 91 (8.8%) were protein-coding genes, lncRNA genes, and pseudogenes, respectively (Figure 2B). We performed a differential gene expression analysis on 475 genes passed by quality control and cell-fraction adjustment. We found that 76.2% (362/475) and 8.4% (40/475) of the genes were significantly upregulated and downregulated in malignant epithelial cells, respectively (Supplementary Figure S8B). Moreover, we found more ACGEJ-gain eQTLs and stronger eQTL effects closer to the TSS of their paired genes (Figures 2C,D). However, we found more ACGEJ-loss eQTLs and stronger eQTL effects at ±300 kb than locations close to the TSS of their paired genes (Figures 2C,D). Furthermore, we found that regulatory effect sizes and statistical power of ACGEJ-gain eQTLs were significantly larger than ACGEJ-loss eQTLs on protein-coding genes, lncRNA genes, or pseudogenes (Supplementary Figures S4A,B,C,D). These findings indicated that either ACGEJ-gain or ACGEJ-loss eQTLs were crucial for ACGEJ susceptibility, while ACGEJ-gain eQTLs might had more power than ACGEJ-loss eQTLs.

### ACGEJ-specific eQTLs potentially regulate expression of cancer-related genes

**FIGURE 3:**
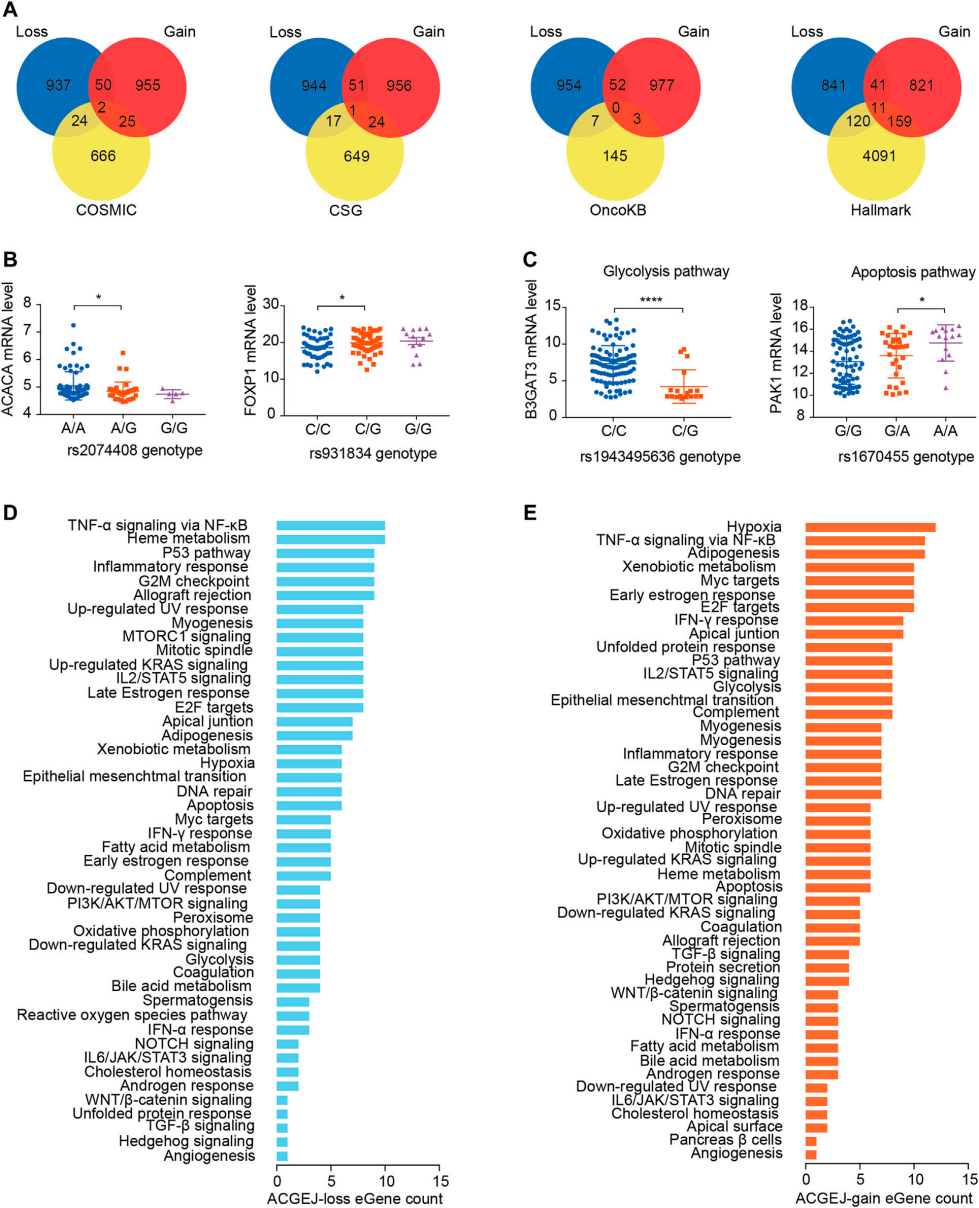
ACGEJ-specific eQTLs acted potentially by regulating the expression of cancer-related genes. **(A)** Interaction analyses between ACGEJ-loss (blue)/ACGEJ-gain (red) genes and cancer-related genes. **(B)** Differential expression of top genes between different genotypes of paired SNPs obtained by COSMIC, CSG, and OncoKB interaction analyses. * and blank represent *p-*value < 0.05 and no significance of the Mann–Whitney test, respectively. **(C)** Differential expression of top genes between different genotypes of paired SNPs within MSigDB hallmark pathways. *, ****, and blank represent *p-*value < 0.05, *p-*value < 0.0001, and no significance of the Mann–Whitney test, respectively. **(D–E)** Distribution of ACGEJ-loss **(D)** and ACGEJ-gain **(E)** genes in each pathway of MSigDB hallmark gene sets. Pathways are ranked by gene counts.

We identified ACGEJ-specific eQTLs that were overlapped with public cancer-related data sets such as the molecular signature database hallmark, catalog of somatic mutations in cancer, cancer susceptibility gene, and MSK’s precision oncology knowledge base. We found 26, 18, 7, and 131 ACGEJ-loss genes in COSMIC, CSG, OncoKB, and Hallmark, respectively (Figure 3A). We also found 27, 25, 3, and 170 ACGEJ-gain genes in COSMIC, CSG, OncoKB, and Hallmark, respectively (Figure 3A). To explore the effect size of particular ePairs, we exhibited the top eight ePairs of which genes were included in the COSMIC, CSG, and OncoKB gene sets sorted by *p*-values. For instance, ACGEJ cells with rs2074408G had significantly lower *ACACA* expression than ones with rs2074408A (Figure 3B). Another example is that ACGEJ cells with rs931834G showed significantly lower *FOXP1* expression than ones with rs931834C (Figure 3B). Furthermore, 131 of the 1,009 (13%) ACGEJ-loss genes and 170 of the 1,027 (16.6%) ACGEJ-gain genes were MSigDB hallmark pathway member genes. We sorted hallmark pathways by the number of genes in them (Figures 3D,E). For instance, ACGEJ cells with rs1943495636G showed significantly lower *B3GAT3* expression, while ACGEJ cells with rs1670455A showed significantly higher *PAK1* expression (Figure 3C).

**FIGURE 4:**
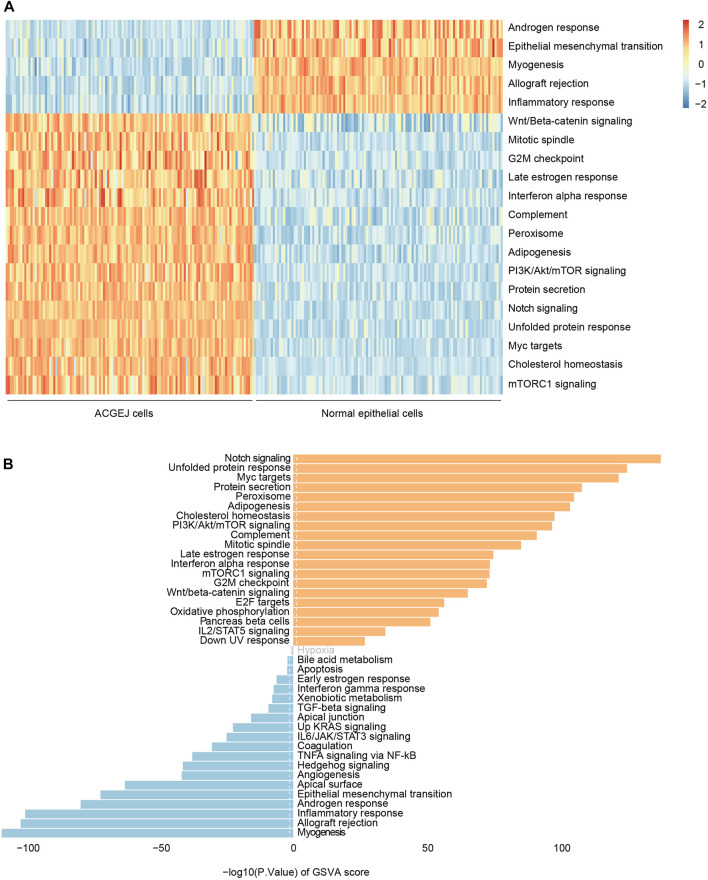
Gene set variation analysis of ACGEJ-specific genes. **(A)** Heatmap of gene set variation analysis of ACGEJ-specific genes. The color depth represents the level of gene expression variation. ACGEJ cells (left); normal epithelial cells (right). **(B)**. Distribution of ACGEJ-loss (blue)/ACGEJ-gain (red) genes in each pathway of MSigDB hallmark gene sets. Pathways are ranked by gene counts.

Since we observed eQTL specificity on cell types (ACGEJ or normal epithelial cells), we performed gene set variation analyses of ACGEJ-loss and ACGEJ-gain genes to explore potential pathway level mechanisms of this condition-specific phenomenon. We found that androgen response, epithelial–mesenchymal transition, myogenesis, allograft rejection, and inflammatory response pathways were significantly downregulated in ACGEJ cells, while pathways such as Wnt/beta-catenin signaling, mitotic spindle, G2-M checkpoint, notch signaling, and unfolded protein response were significantly upregulated in ACGEJ cells (Figure 4A). Furthermore, notch signaling, unfolded protein response, and Myc targets were the top three upregulated pathways, while myogenesis, allograft rejection, and inflammatory response were the top three downregulated pathways (Figure 4B).

### rs658524G>A mutation reduces *CTSW* expression by reinforcing KLF5 binding ability

**FIGURE 5:**
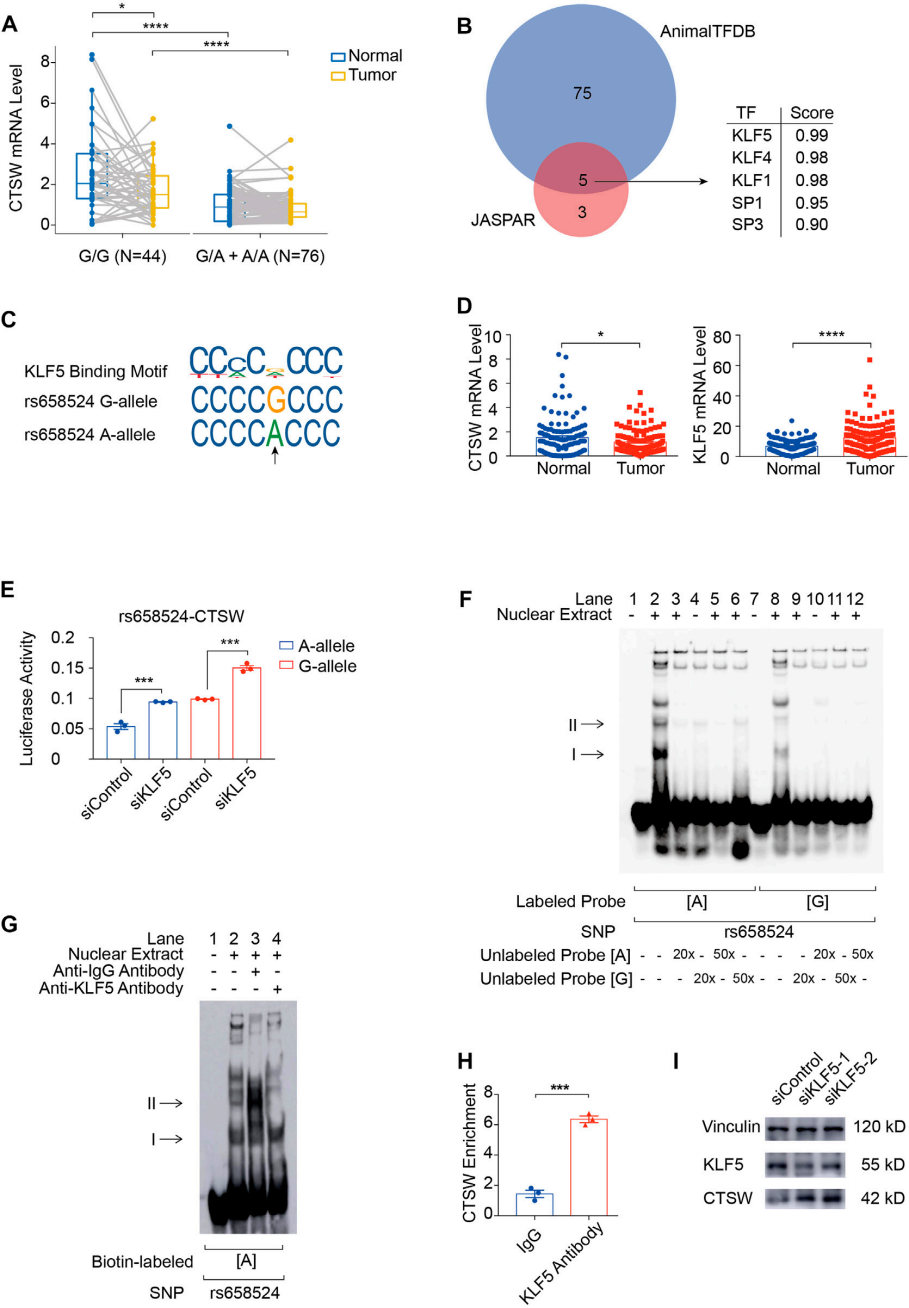
rs658524G>A mutation reduces *CTSW* expression via KLF5. **(A)** Differential expression of *CTSW* expression between normal epithelial cells and ACGEJ cells. Samples are divided by genotypes. *, ****, and blank represents *p-*value < 0.05, *p-*value < 0.0001, and no significance of the Wilcoxon test, respectively. **(B)** Potential transcription factors of *CTSW* that might bind to rs658524 are predicted by JASPAR and AnimalTFDB affinity score using DNA fragments containing rs658524G (25 base pairs from the position −84 to −60). **(C)** JASPAR and HumanTFDB are used to predict the binding site of KLF5 at rs658524. **(D)** Differential expression of *CTSW* and *KLF5* between normal epithelial cells and ACGEJ cells. **(E)** pGL3-promoter plasmids are transfected into OE19 24 h after the transfection of siRNA of *KLF5*. Relative luciferase activity is examined 24 h after the transfection of plasmids with triple repetition. *** represents *p-*value < 0.001 of the unpaired *t*-test. **(F)** EMSA assay of rs658524. Concentration of unlabeled probes is designed to be 20- or 50-fold of biotin-labeled probes. **(G)** Super-shift EMSA assay of rs658524 using the KLF5 antibody. **(H)** ChIP-qPCR assay of rs658524 using the KLF5 antibody and rabbit IgG with triple repetition. *** represents *p-*value < 0.001 of the unpaired *t*-test. **(I)** Western blot is performed 48 h after the transfection of siRNA of *KLF5*.

To explore regulatory functions of ACGEJ-specific eQTLs, we annotated these eQTLs with their regulatory distance between each eQTL and transcription start site of their paired gene and found that ACGEJ-gain eQTLs were enriched at promoter regions (Figures 2C,D). We then performed functional analyses on the top eQTL rs658524 of which the distance between rs658524 and the transcription start site of *CTSW* was 71 base pairs. Bayesian fine-mapping analysis revealed that rs658524 was the potential causal eQTL of *CTSW* (Supplementary Table S9). We performed differential expression analysis on adjusted *CTSW* expression and found that the *CTSW* mRNA level of samples with rs658524G/G was significantly higher than for ones with rs658524G/A and rs658524A/A in normal epithelial cells (Figure 5A). However, we found no significant difference in *CTSW* expression between samples with rs658524G/G and the ones with rs658524G/A and rs658524A/A (Figure 5A). Furthermore, we found that *CTSW* expression in normal epithelial cells were significantly higher than that in ACGEJ cells (Figures 5A,C), suggesting *CTSW* was inhibited during ACGEJ initiation or progression. We predicted potential transcription factors of *CTSW* by the affinity score and regarded KLF5 as the top potential transcription factors of *CTSW* binding to rs658524 (Figures 5B,C). The adjusted expression of *KLF5* in normal epithelial cells was significantly lower than ACGEJ cells, suggesting that *KLF5* might suppress the transcription of *CTSW* as an oncogene (Figure 5D). To explore the influence of *KLF5* on *CTSW* transcription, we first performed the reporter assay. We indicated that rs658524G>A suppressed relative luciferase activity while the knockdown of *KLF5* rescued this suppression (Figure 5E). Additional EMSA assays showed that the nuclear protein bound to the DNA probe was rs658524A specific, and the band could be super-shifted when the KLF5 antibody was included in the incubation mixture (Figures 5F,G), indicating that the protein bound to rs658524 was likely to be KLF5. ChIP-qPCR detection in the OE19 cell line with the rs658524A/A genotype (Supplementary Figure S5A,B) showed significantly stronger KLF5 binding ability to the rs658524 region than rabbit IgG (Figure 5H). Knockdown of *KLF5* promoted *CTSW* expression in the OE19 cell line (Figure 5I). Taken together, these results demonstrate that rs658524G>A mutation promoted KLF5 binding ability that caused *CTSW* downregulation in ACGEJ where *KLF5* was overexpressed.

### Identification of ACGEJ-specific susceptibility and prognosis markers

**FIGURE 6:**
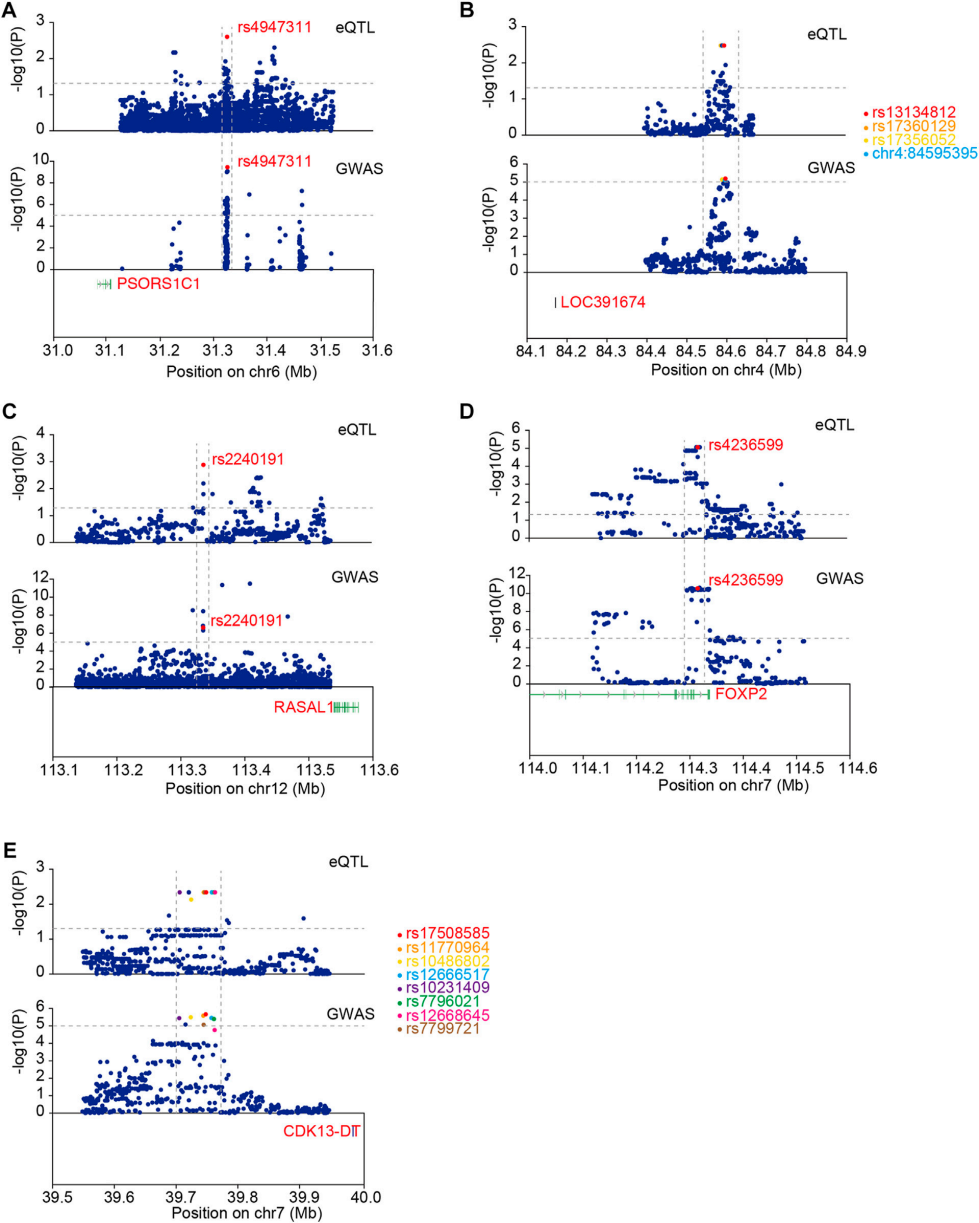
Co-localization analysis of ACGEJ-specific eQTLs and GWAS loci. **(A)** eQTLs: upper; GWAS: lower. The horizontal dotted lines represent *p-*value ≤ 0.05 (upper) and *p-*value ≤ 0.00001 (lower). The vertical dotted lines represent the same peak both in eQTL and GWAS data set. Susceptibility gene *PSORS1C1* is highlighted in red. **(B)** eQTLs: upper; GWAS: lower. The horizontal dotted lines represent *p-*value ≤ 0.05 (upper) and *p* ≤ 0.00001 (lower). Susceptibility gene *LOC391674* is highlighted in red. Top 10 eQTLs within GWAS loci are highlighted. **(C)** eQTLs: upper; GWAS: lower. The horizontal dotted lines represent *p-*value ≤ 0.05 (upper) and *p-*value ≤ 0.00001 (lower). Susceptibility gene *RASAL1* is highlighted in red. **(D)**. eQTLs: upper; GWAS: lower. The horizontal dotted lines represent *p-*value ≤ 0.05 (upper) and *p-*value ≤ 0.00001 (lower). Susceptibility gene *FOXP2* is highlighted in red. **(E)** Susceptibility gene *CDK13-DT* is highlighted in red. Top 10 eQTLs within GWAS loci are highlighted.

We performed co-localization analysis of ACGEJ-specific eQTLs and GWASs to explore the contribution of these eQTLs to ACGEJ susceptibility. We identified five eQTLs co-localized with identical GWAS loci, namely, rs4947311–*PSORS1C1* (PP4 = 0.32) (Figure 6A), rs13134812–*LOC391674* (PP4 = 0.25) (Figure 6B), rs2240191–*RASAL1* (PP4 = 0.32) (Figure 6C), rs4236599–*FOXP2* (PP4 = 0.41) (Figure 6D), and rs17508585–*CDK13-DT* (PP4 = 0.29) (Figure 6E, Supplementary Table S6). We also found that distribution patterns of ACGEJ-specific eQTLs were consistent. An example was rs13134812—*LOC391674*, of which the peak of ACGEJ-specific eQTLs and GWASs appeared at the same zone. Notably, rs4236599–*FOXP2* was also exhibited in two GWAS datasets with similar patterns (Supplementary Figure S6A). Furthermore, rs2240191–*RASAL1* showed up in two GWAS data sets with similar patterns (Supplementary Figure S6B). Notably, functions of these genes might be crucial for ACGEJ susceptibility and worthy of further study.

**FIGURE 7:**
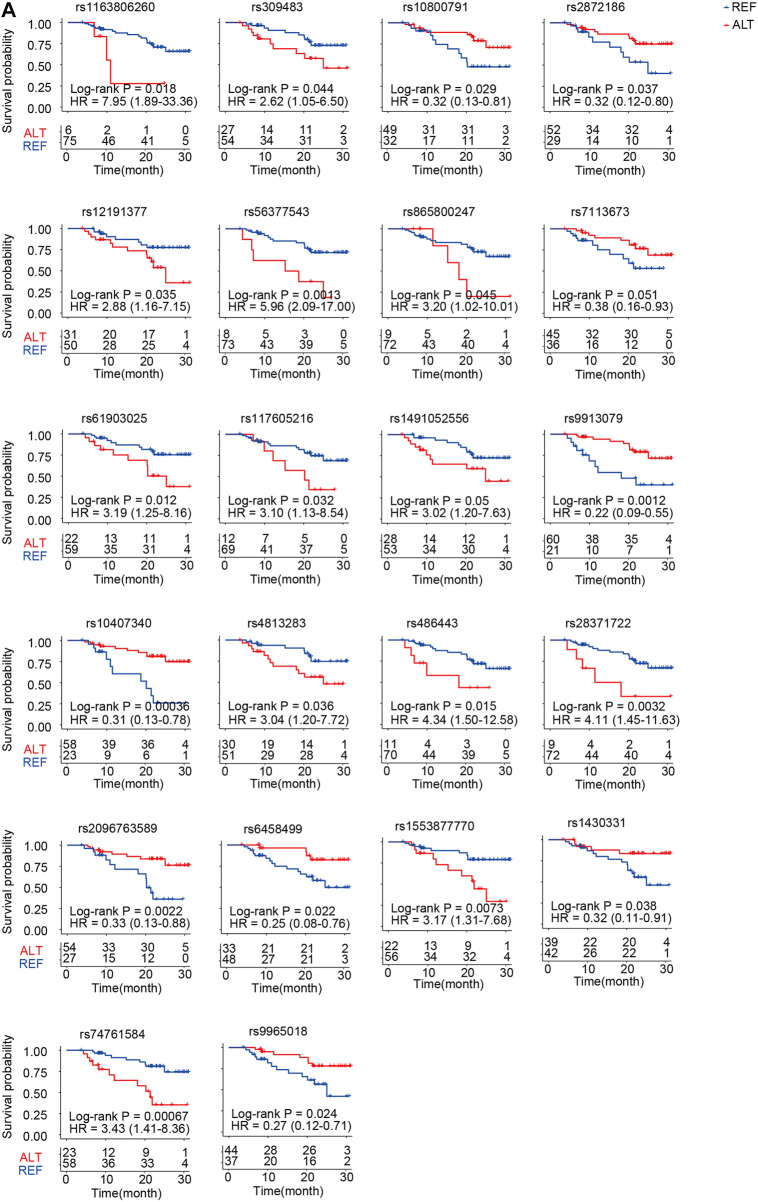
Twenty-two non-coding prognosis markers. **(A)** Kaplan–Meier survival curves for patients with different genotypes of SNPs that potentially regulate non-coding RNA expression.

We further performed survival analysis of ACGEJ-specific eQTLs. In total, 55 eQTLs were significantly associated with overall survival by the log-rank test and the Cox proportional hazards model adjusted by gender, age, smoking status, drinking status, and TNM stages (Supplementary Table S7). Nearly half (22/55) of the genes paired to their survival-associated eQTLs were non-coding genes, that is, 10 pseudogenes and 9 lncRNA genes (Figure 7A). An example was rs10407340–vomeronasal 1 receptor pseudogene. Patients with rs10407340G (homozygote alteration genotype and heterozygote) had better survival than those with rs10407340A (homozygote reference genotype) (log-rank *p* = 0.00036, HR = 0.31, and 95% CI = 0.13–0.78) (Figure 7A). We also visualized overall survival with other genes except for these 22 non-coding genes (Supplementary Figure S7A). Furthermore, we performed survival analyses of genes paired with these survival-associated eQTLs. Patients with rs309483G had better survival than those with rs309483A with log-rank *p* being 0.04 and HR being 2.62 (1.05–6.50). We found that patients with high-level *LINC01355* (rs309483 paired gene) had better overall survival than those with low-level *LINC01355* [*p* = 0.005 and HR = 0.34 (0.16–0.73)], which is contrary to the Kaplan–Meier plotter result [log-rank *p* = 0.00092 and HR = 1.44 (1.16–1.79)]. The effect size of rs309483 on *LINC01355* expression was −2.76, indicating that germline alteration rs309483G>A might downregulate *LINC01355* expression and better survival of rs309483G than rs309483A and suggesting the potential of rs309483–*LINC01355* as an ACGEJ-specific prognosis marker.

**FIGURE 8:**
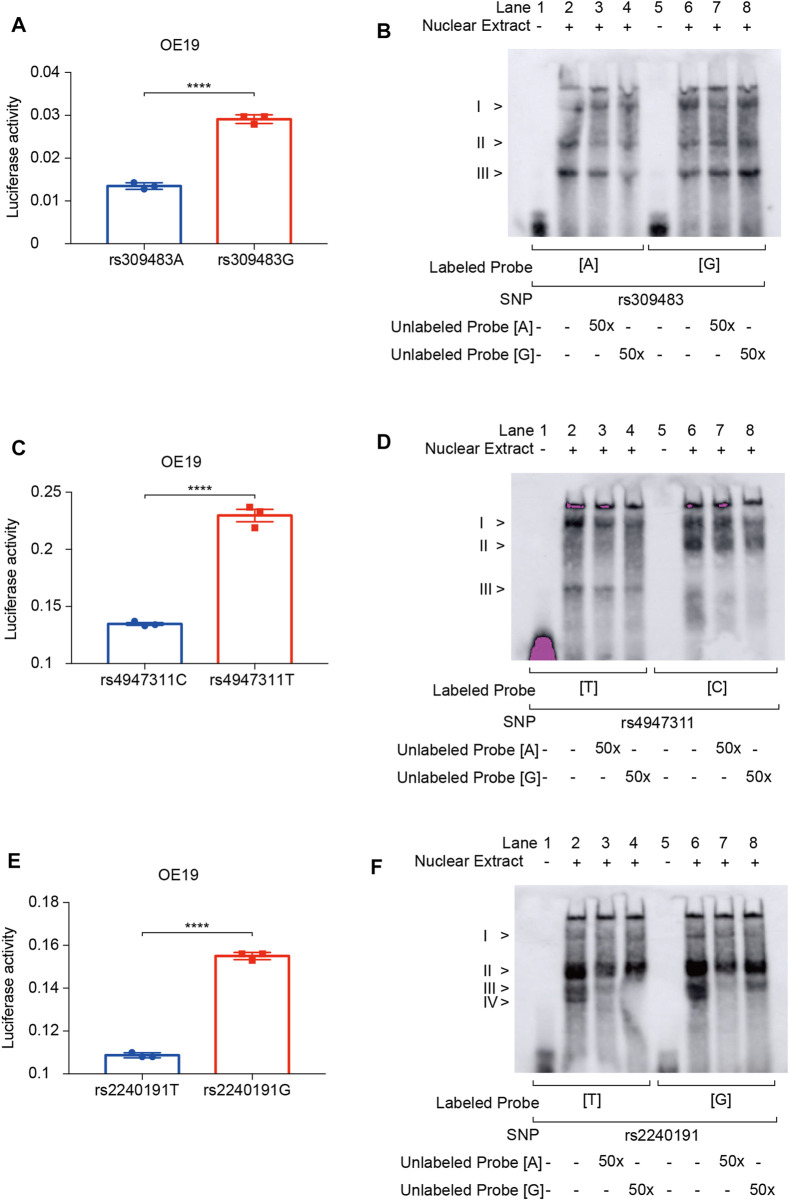
Reporter gene and EMSA assays of survival and susceptibility loci. **(A)** Reporter gene assays of rs309483. **(B)** EMSA assays of rs309483; concentration of unlabeled probes is set to be 50-fold of biotin-labeled probes. **(C)** Reporter gene assays of rs4947311. **(D)** EMSA assays of rs4947311; concentration of unlabeled probes is set to be 50-fold of biotin-labeled probes. **(E)** Reporter gene assays of rs2240191. **(F)** EMSA assays of rs2240191; concentration of unlabeled probes is set to be 50-fold of biotin-labeled probes.

To explore the functions of these susceptibility and prognosis markers in ACGEJ, we performed reporter gene assays and found that rs309483A>G (*LINC01355*-paired eQTL) (Figure 8A), rs4947311C>T (*PSORS1C1*-paired eQTL) (Figure 8C), and rs2240191T>G (*RASAL1*-paired eQTL) promoted relative luciferase activity (Figure 8E). EMSA assays indicated no less than three nuclear proteins that bound to rs309483 (Figure 8B), rs4947311 (Figure 8D), and rs2240191 (Figure 8F), suggesting their roles as enhancers during the transcription of *LINC01355*, *PSORS1C1*, and *RASAL1*, respectively.

## Discussion

Over the last decades, millions of eQTLs have been identified using bulk RNA-seq and WGS data to explore critical biological events of complex diseases (Li et al., 2013; Zhu et al., 2016; Tachmazidou et al., 2019). More and more eQTL studies have confirmed that regulatory patterns of gene expression exhibit specificity in particular cell types (Raj et al., 2014; Schmiedel et al., 2018; Young et al., 2021). However, cell type–specific eQTLs still remain scarce in tumor studies. In the present study, we promoted eQTL resolution on cell type level by leveraging scRNA-seq, bulk RNA-seq, and WGS data (Westra et al., 2015; Geeleher et al., 2018; Donovan et al., 2020). As a result, we obtained 2,036 ACGEJ-specific eQTLs that might consist of real regulatory information in ACGEJ. Previous studies have confirmed important roles of non-coding RNA genes as clinical or prognosis markers in initiation or progression of solid tumors (Li et al., 2020; Xin et al., 2021). However, most RNA-seq data obtained by capturing polyA of mRNA lead to poor coverage on non-coding RNAs. In the present study, we found that approximately 25% of the ACGEJ-specific genes were lncRNA genes and pseudogenes, suggesting their huge potential as ACGEJ-specific therapeutic or prognosis markers. The GTEx project has revealed tissue specificity of eQTLs in normal samples (Battle et al., 2017). We found that 0.6% of our results overlapped with gastroesophageal junction–specific eQTLs of the GTEx database. Notably, the sampled donors of the GTEx project (v7) were 83.7% European Americans and 15.1% African Americans, suggesting that our results were ancestry specific.

Cell type–specific eQTL findings reveal regulatory heterogeneity between normal and malignant epithelial cells at the gastroesophageal junction. These ACGEJ-specific eQTL results may be due to genomic instability, epigenetic reprogramming, and the tumor micro-environment (TME). Genomic instability such as copy number variants (CNV) and somatic mutations may lead to the activation of oncogenes and inactivation of tumor suppressor genes. Based on a previous study of genomic and transcriptomic alterations, ACGEJ in Chinese patients have been profiled as a CNV-dominant tumor (Lin et al., 2020). Driver genes that may play oncogenic roles, such as *CCNE1*, *ERBB2*, *VEGFA*, and *RICTOR*, were amplified, whereas driver genes that may act as suppressors, such as *CDKN2B*, *MTAP*, *PTEN*, and *FAT1*, were deleted both in the ACGEJ and TCGA cohorts (Lin et al., 2020). Transcription factors that were differentially expressed may also lead to these ACGEJ-specific eQTL results. We performed differential gene expression analysis of 660 transcription factors, in which the mRNA levels were adjusted by using CIBERSORTx and filtered by quality control between tumor and normal samples. Of all passed transcription factors, 75.3% (497/660) and 8.5% (56/600) were upregulated and downregulated, respectively (Supplementary Figure S8A). Epigenetic reprogramming could contribute to DNA accessibility alteration during tumorigenesis and progression (Hanahan, 2022). Previous studies have indicated that the epigenetic silencing of promoter regions of genes such as *RASSF1A*, *TSP1*, *INK4A*, and *FBXO32* influenced the progression of ACGEJ (Abdi et al., 2019). The TME of gastric cancer has been indicated to be enriched for stromal cells, macrophages, dendritic cells, and Tregs (Sathe et al., 2020). Cellular stress from the TME, such as cancer-associated fibroblasts (CAFs), has been indicated to influence crucial gene expression in gastric cancers to increase sensitivity to chemotherapeutic treatment (Yang et al., 2022).

To explore the key pathways of ACGEJ-specific genes, we performed the gene set variation analysis on ACGEJ-specific genes and found that many cancer hallmark pathways were significantly up or downregulated in ACGEJ cells. The top upregulated and downregulated pathways were notch signaling and myogenesis pathways, respectively. The Notch signaling pathway has been confirmed to be upregulated in Barrett-like metaplasia and necessary for maintaining the gastric stem cell compartment (Kim and Shivdasani, 2011; Quante et al., 2012). Pro-inflammatory cytokines produced by tumor cells have been indicated to directly impact myogenesis in several ways (Fearon et al., 2012).

Previous studies have confirmed that eQTLs probably regulate genes through TFs (Gaffney et al., 2012; Flynn et al., 2022), therefore we focused on the potential transcription factors of ACGEJ-specific genes. We found that rs658524G>A mutation suppressed *CTSW* expression by reinforcing the binding ability of KLF5, suggesting that genomic alteration and overexpressed transcription factors regulated key genes together.

Previous GWASs toward esophageal and gastric cancer have identified hundreds of susceptible genes and loci (Buas et al., 2017; Jin et al., 2020). However, the relationship between these loci and nearby genes still remains unclear. In the present study, we undertook co-localization analysis and found five susceptible ePairs of eQTLs and genes sharing good consistence with previous GWAS results. We then performed survival analysis of ACGEJ-specific eQTLs. Notably, nearly half of the survival-associated genes were non-coding genes. For example, previous studies have demonstrated the important role of *LINC01355* as a tumor suppressor or activator gene (Ai et al., 2019; Piao et al., 2022). A high level of *LINC01355* led to poor survival of gastric cancer patients as shown in previous studies (Ai et al., 2019) and the Kaplan–Meier plotter database, which is contrary to our results. Moreover, *LINC01355* could be significantly inhibited by drugs such as tozasertib, docetaxel, and Belinostat (Nath et al., 2019), suggesting the great potential of rs309483–*LINC01355* being an ACGEJ-specific survival and therapeutic marker. We explored functions of rs309483, rs2240191, and rs4947311, suggesting their potential role as enhancers. Furthermore, we explored the functions of *LINC01355*, *RASAL1*, and *PSORS1C1* in the ACGEJ cell line OE19, revealing their important roles as oncogenes or suppressor genes in OE19. Notably, future studies should consider the contribution of eQTLs in non-coding regions to ACGEJ progression. Such research studies, combined with our findings, have the potential to inform drug development, risk assessment, and clinical practice toward reducing the burden of ACGEJ.

Nevertheless, it is important to acknowledge some limitations of our study. First, we performed cis-eQTLs not trans-eQTLs because of our relatively small sample size. Studies have further demonstrated quite different regulatory patterns of trans-eQTLs from cis-eQTLs (Liu et al., 2019; Võsa et al., 2021). Contrary to cis-eQTLs, trans-eQTLs were more likely to impact core gene expression through peripheral genes mediately. Second, the adjusted mRNA level of low-expressed genes (TPM < 1) might not be accurate enough, which may influence the adjustment of partial non-coding genes.

## Conclusion

Our study identified 1,009 ACGEJ-loss and 1,027 ACGEJ-gain eQTLs, nearly one-fourth of which were associated with the gene expression of non-coding genes. We also elucidated the enrichment of ACGEJ-specific genes in cancer hallmark pathways such as notch signaling and myogenesis. Finally, we identified five potential susceptibility and one prognosis marker of ACGEJ. Our study provides new insights into the impact of ACGEJ-specific germline mutations on irregular gene expression, reinforces the importance of non-coding genes playing roles in ACGEJ susceptibility, and may shed light on the prognosis of ACGEJ patients.

## Data Availability

The data sets presented in this study can be found in online repositories. The names of the repository/repositories and accession number(s) can be found in the article/Supplementary Material.
