# Supplementary figures and images for: Refined expression quantitative trait locus analysis on adenocarcinoma at the gastroesophageal junction reveals susceptibility and prognostic markers
Source: Front Genet. 2023 May 17;14:1180500. doi: 10.3389/fgene.2023.1180500 (PMC10230079; doi:10.3389/fgene.2023.1180500)

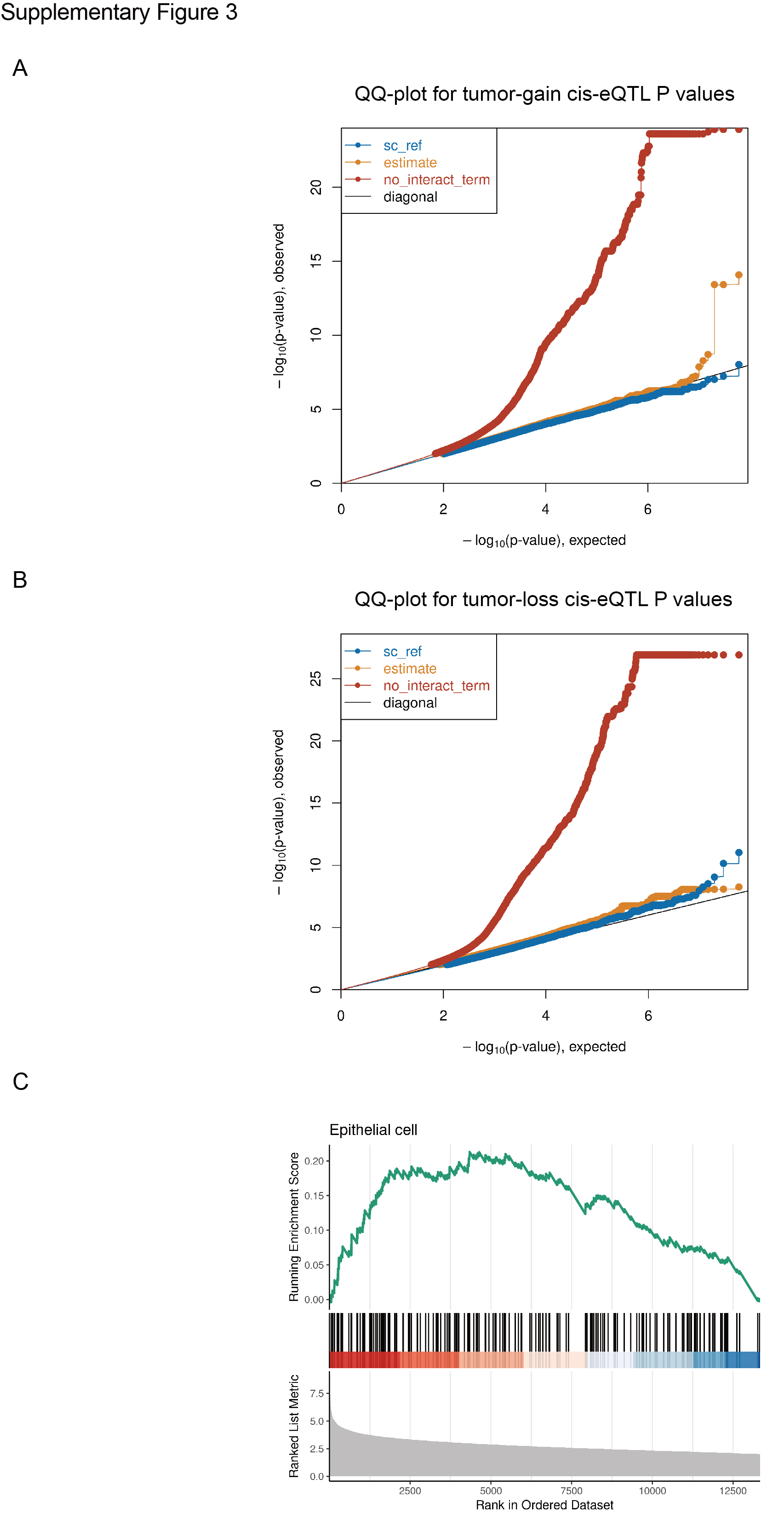

Supplement: Supplementary file 2 [file Image3.JPEG]

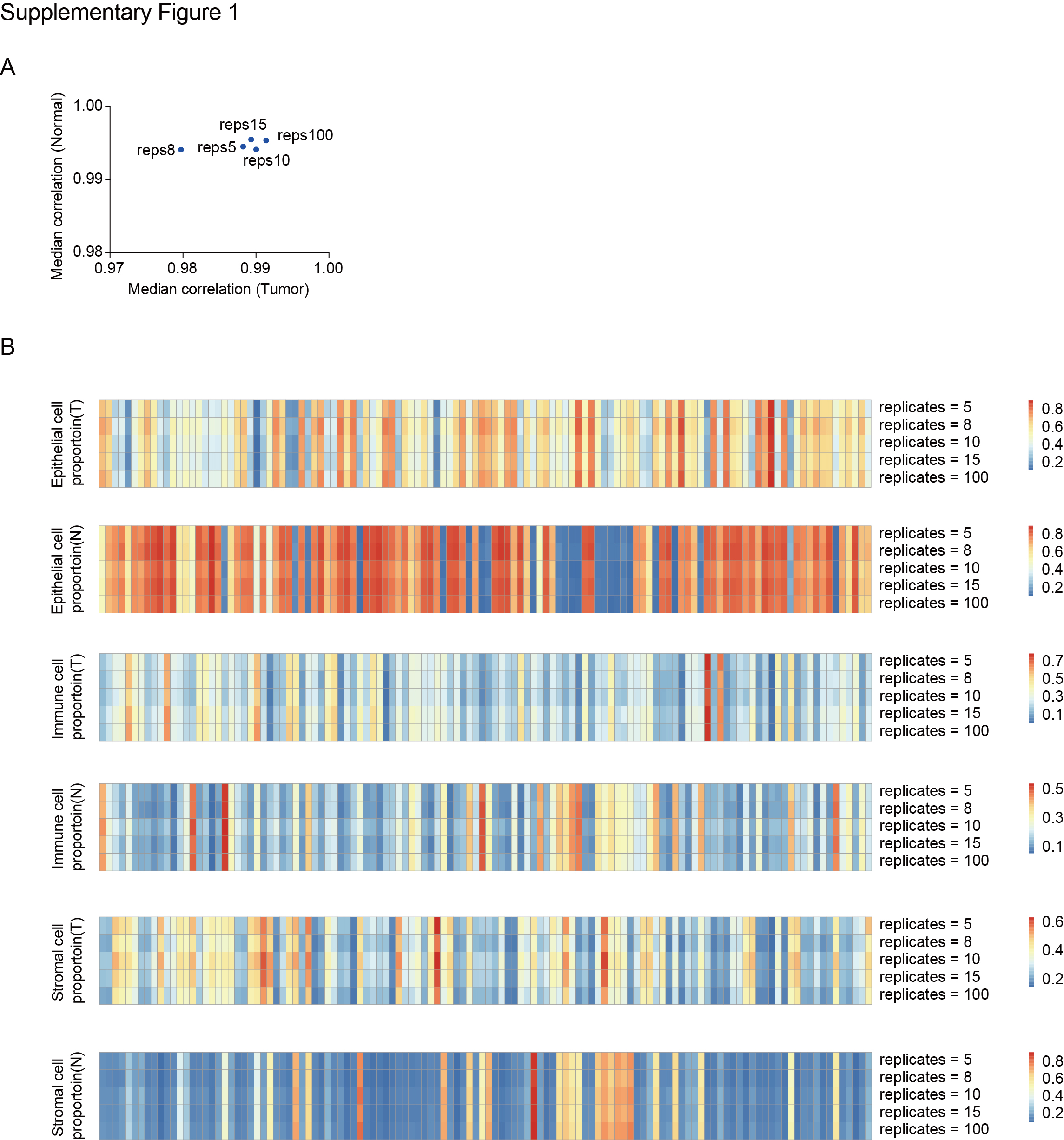

Supplement: Supplementary file 4 [file Image1.JPEG]

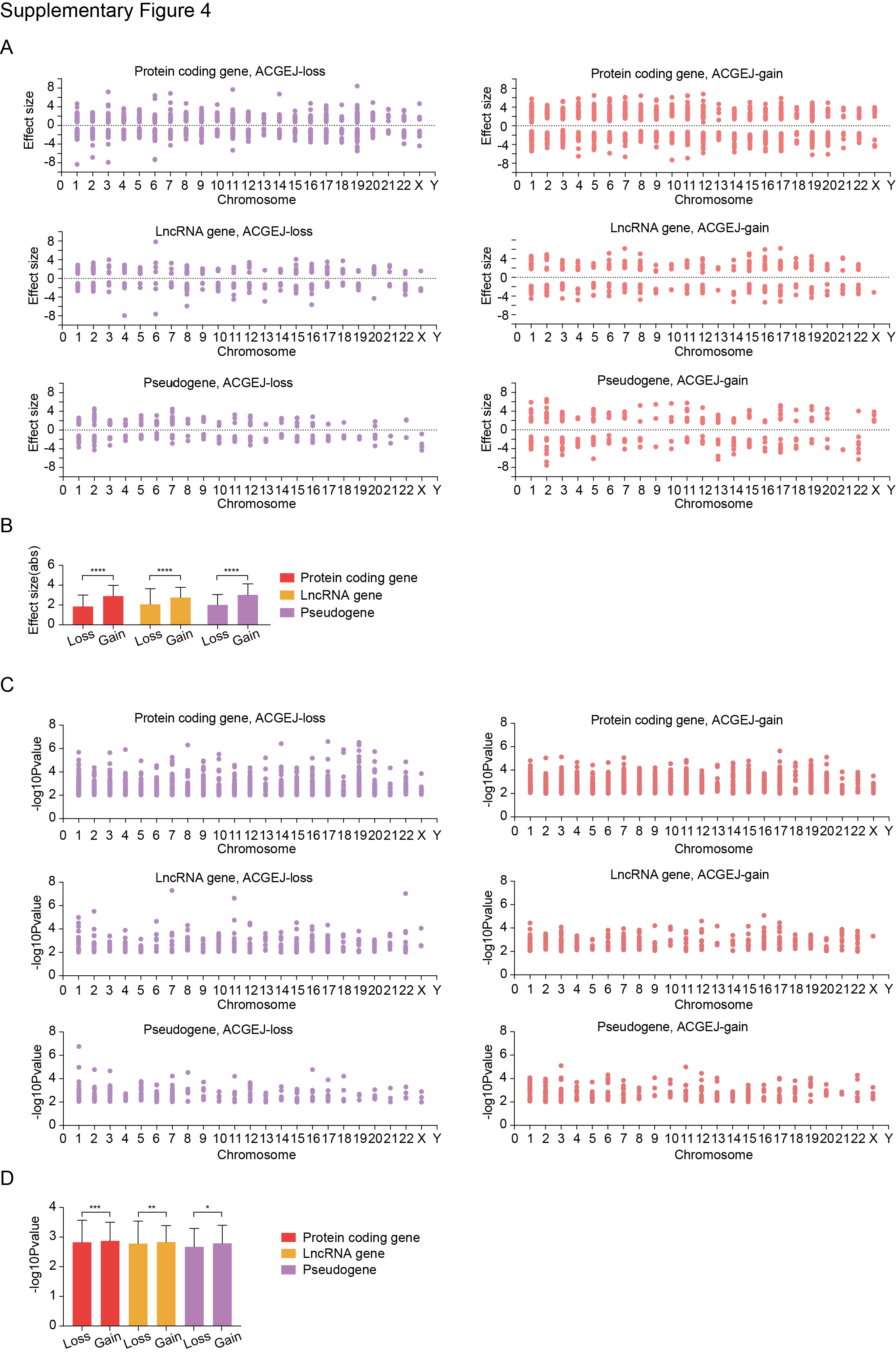

Supplement: Supplementary file 5 [file Image4.JPEG]

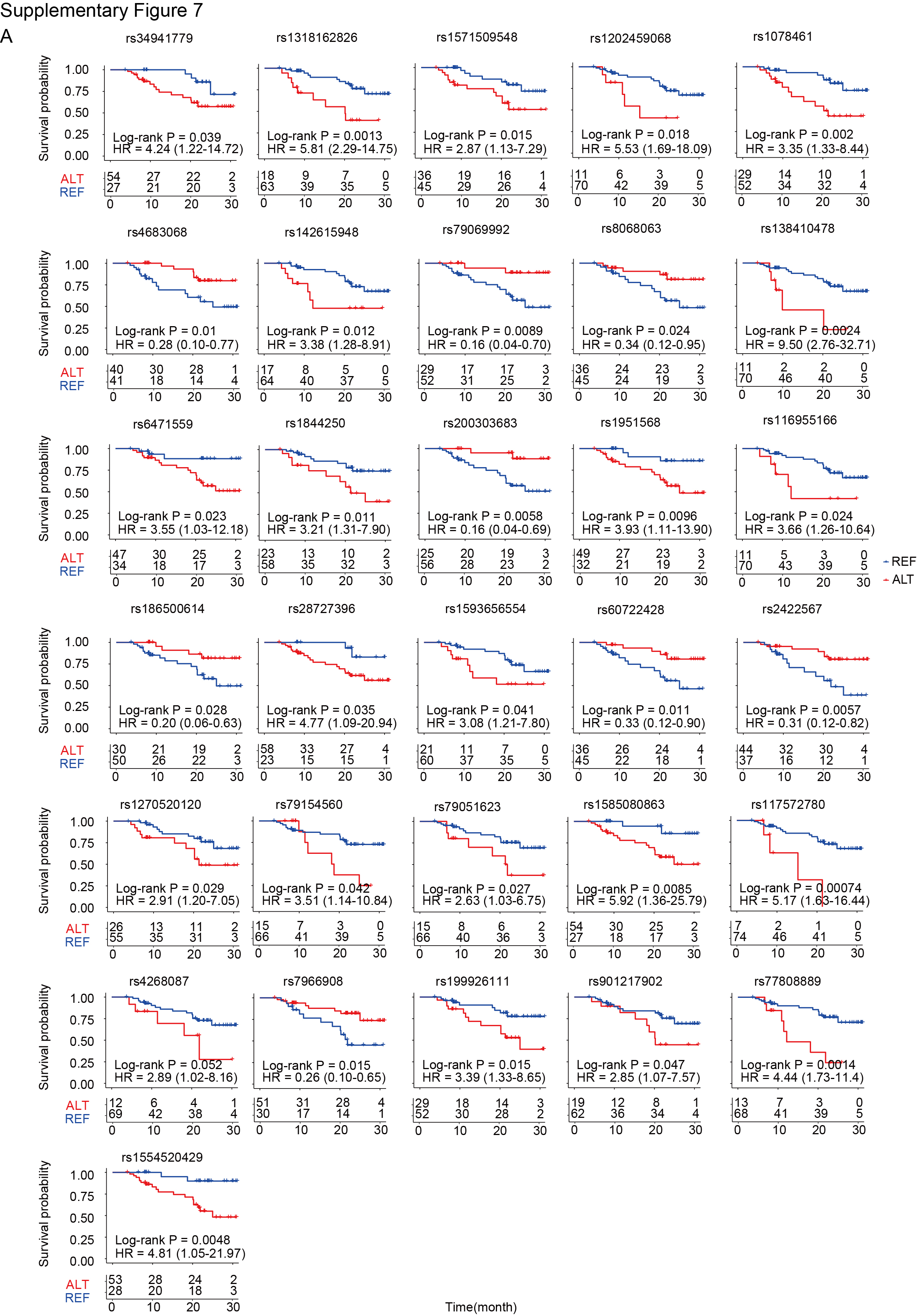

Supplement: Supplementary file 6 [file Image7.JPEG]

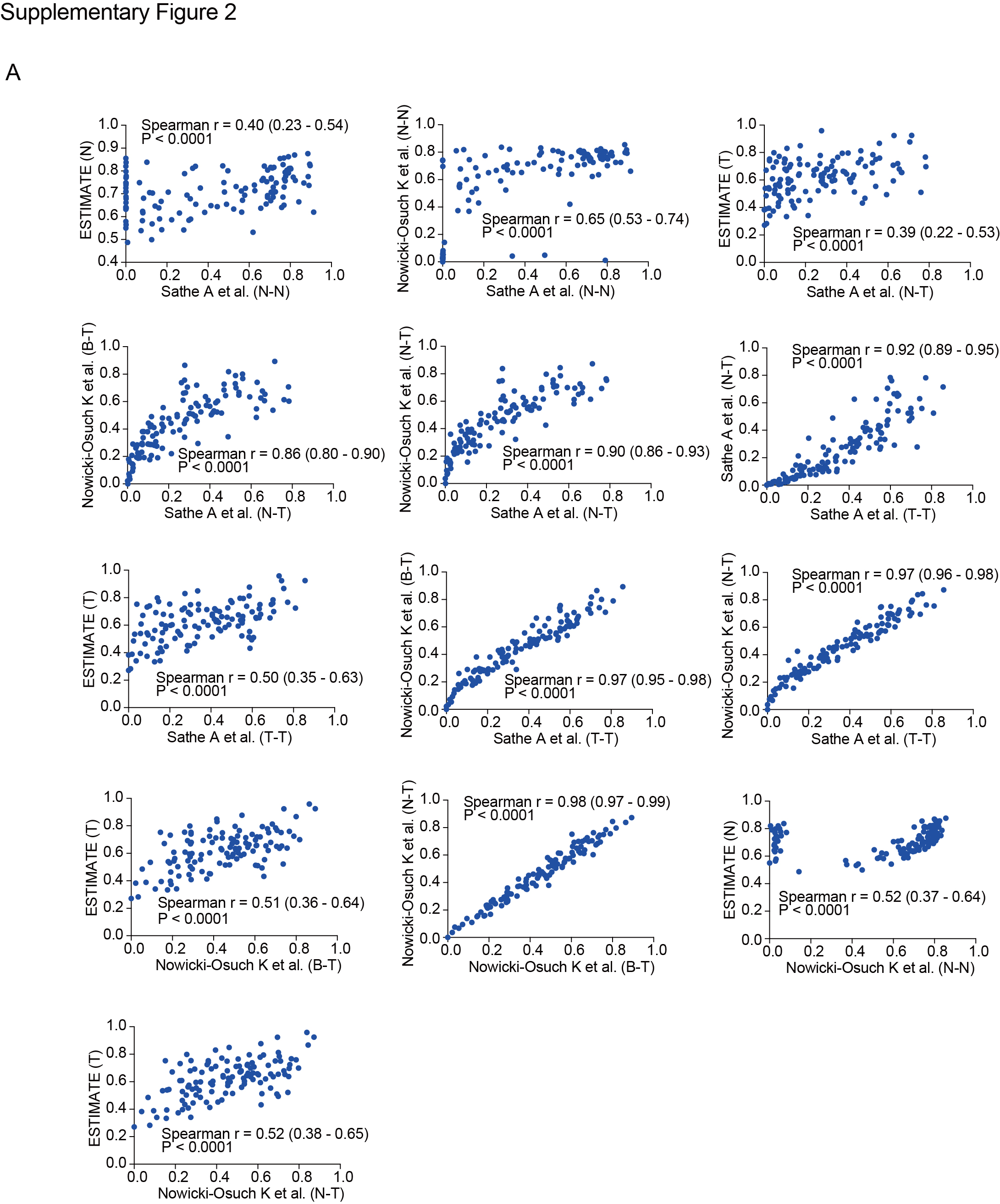

Supplement: Supplementary file 7 [file Image2.JPEG]

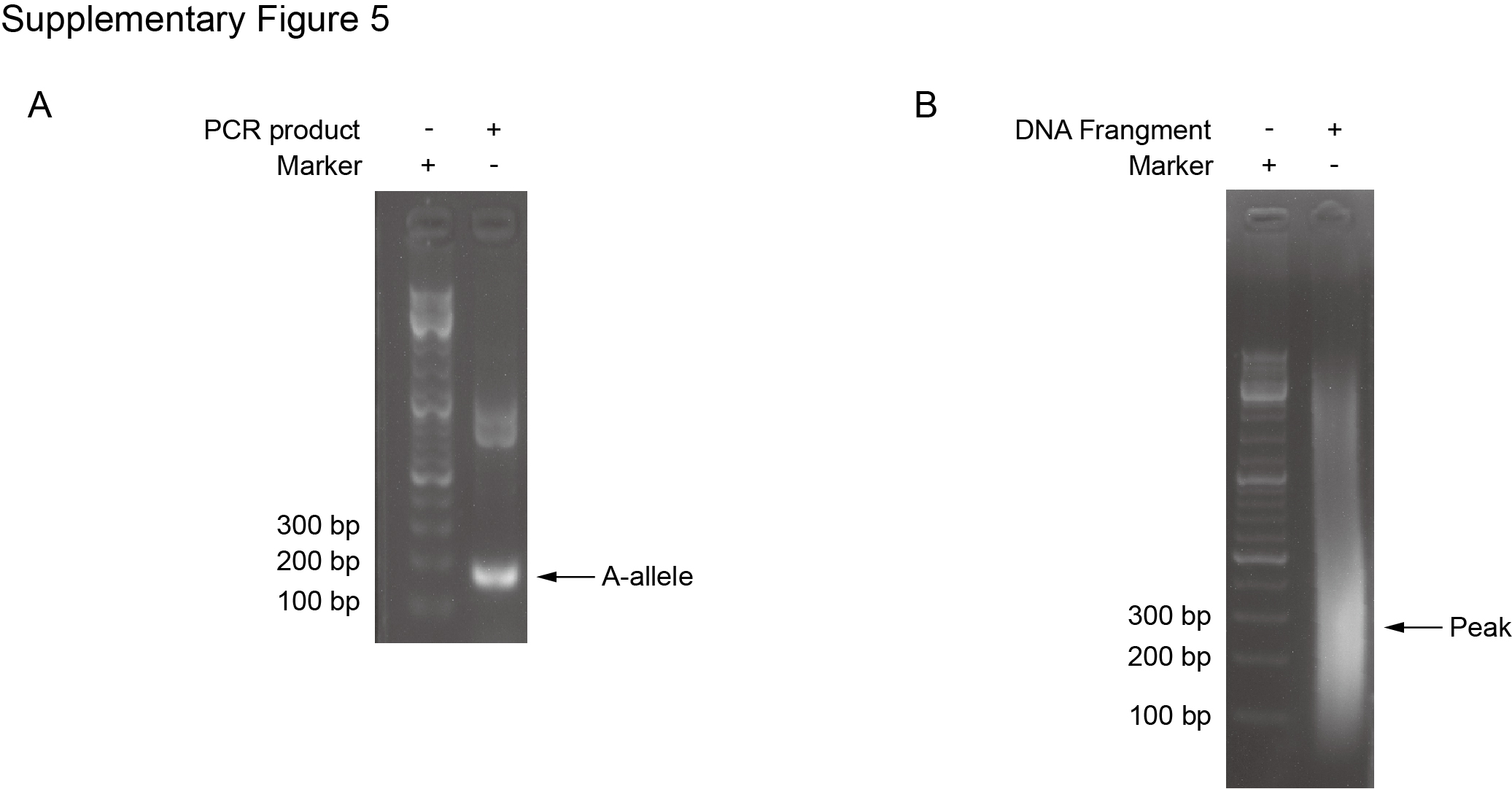

Supplement: Supplementary file 8 [file Image5.JPEG]

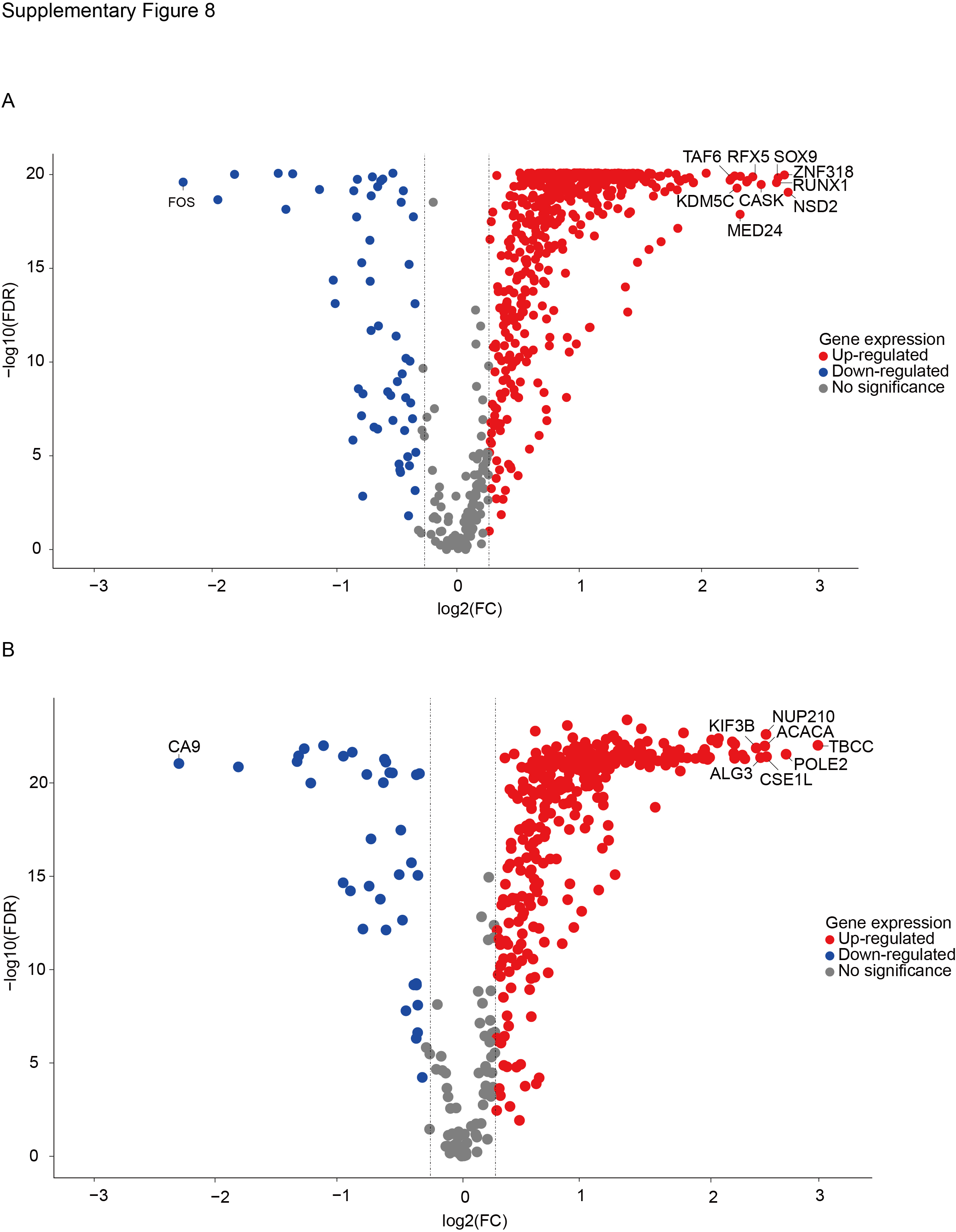

Supplement: Supplementary file 9 [file Image8.JPEG]

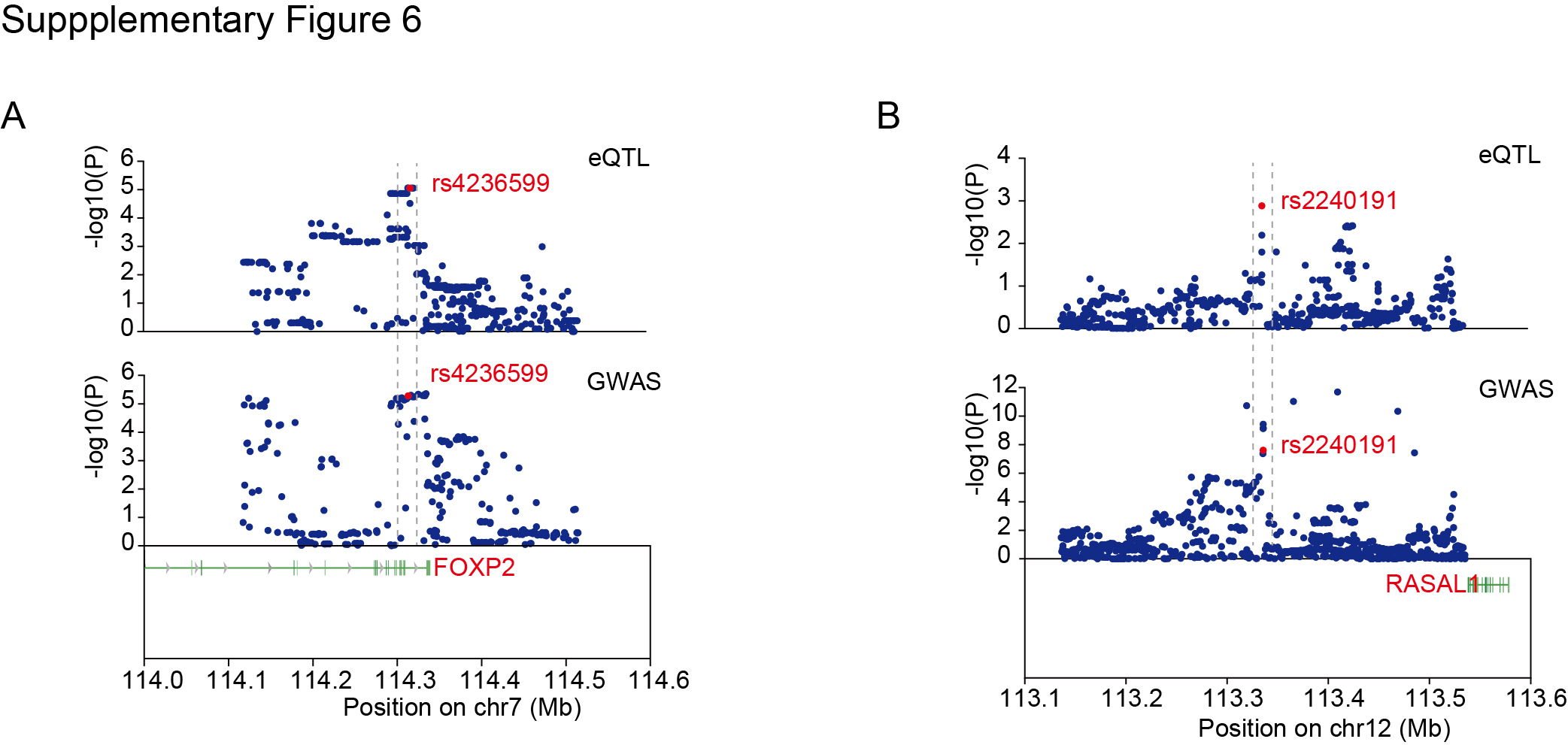

Supplement: Supplementary file 10 [file Image6.JPEG]
