# Supplementary material for: Refined expression quantitative trait locus analysis on adenocarcinoma at the gastroesophageal junction reveals susceptibility and prognostic markers
Source: Front Genet. 2023 May 17;14:1180500. doi: 10.3389/fgene.2023.1180500 (PMC10230079; doi:10.3389/fgene.2023.1180500)
Supplement: Supplementary file 3 [file Table1.DOCX]

**SUPPLEMENTARY FIGURE LEGENDS**

**Supplementary Figure 1. Cell proportions with different parameter “replicates” are close.** **(A)** The correlation coefficient between cell proportions of 120 samples was calculated by Spearman correlation test. The X-axis and Y-axis represent the median of all correlation coefficients between one and other parameters in tumor and normal cells, respectively. Parameters include 5 (default), 8, 10, 15 and 100. **(B)** Epithelial, immune and stromal cell proportions of all samples with different replicates (5, 8, 10, 15 and 100) in tumor and normal tissues. The X-axis in heatmaps represents samples and the Y-axis represents parameter ‘replicates’.

**Supplementary Figure 2. Epithelial cell proportions using scRNA-seq data of normal epithelial cells of Nowicki-Osuch K et al. was highly correlated with cell proportions calculated by ESTIMATE.** **(A)** Correlation between epithelial cell proportions of tumor (X-T) or normal (X-N) tissues estimated by ESTIMATE or CIBERSORTx using scRNA-seq data of Sathe A et al. or Nowicki-Osuch K et al. as reference expression files. Correlation coefficients were obtained by Spearman correlation test. B represents Barret’s esophagus, N represents normal and T represents tumor. The X-axis or Y-axis represents arithmetic average of correlations between each two cell proportions estimated by ESTIMATE or CIBERSORTx in tumor or normal tissues, respectively. Correlation between epithelial cell proportions using different data and tools. Sathe A et al. (T-T) means we use gene expressions of epithelial cell scRNA-seq data as the reference file of CIBERSORTx to deconvolute epithelial cell proportion of RNA-seq data of ACGEJ tissues.

**Supplementary Figure 3. Quality control of epithelial cell and ACGEJ-specific eQTLs.** **(A)** QQ-plots of tumor-gain eQTLs P values. The X-axis represents expected -log10(P values) and the Y-axis represents observed -log10(P values). **(B)** QQ-plots of tumor-loss eQTLs P values. The X-axis represents expected -log10(P values) and the Y-axis represents observed -log10(P values). **(C)** GSEA enrichment analysis of epithelial cell-specific eQTLs.

**Supplementary Figure 4. Comparison of effect sizes of ACGEJ-loss and -gain eQTLs .** **(A)** Manhattan plot of ACGEJ-loss (left, purple) and -gain eQTLs (right, red) clustered by functions of paired genes. X- and Y-axis represents chromosome and effect size, respectively. **(B)** Differential effect size analysis between ACGEJ-loss and -gain eQTLs. **** represents P < 0.0001 of Mann-Whitney test. **(C)** Manhattan plot of ACGEJ-loss (left, purple) and -gain eQTLs (right, red) clustered by functions of paired genes. X- and Y-axis represents chromosome and adjusted P value, respectively. **(D)** Differential adjusted P value analysis between ACGEJ-loss and -gain eQTLs. *, ** and *** represents P < 0.05, P < 0.01 and P < 0.001 of Mann-Whitney test.

**Supplementary Figure 5. Genotype validation and ChIP-qPCR quality control.** **(A)** Genotype of rs658524 of ACGEJ cell line OE19. **(B)** DNA fragments used in ChIP-qPCR assay.

**Supplementary Figure 6. Co-localization analysis of ACGEJ-specific eQTLs and GWAS loci.** **(A)** ACGEJ-specific eQTL rs4236599 (red dot) was identified as top locus both in epithelial cell-specific eQTLs (upper) and GWAS (lower) data set GCST90000515. The horizontal dotted lines represented P ≤ 0.05 (upper) and P ≤ 0.00001 (lower). The vertical dotted lines represented the same peak both in eQTL and GWAS data set. Genes from 114.0 Mb to 114.6 Mb on chromosome 1 were showed up in the box and susceptibility gene FOXP2 was highlighted in red. **(B)** ACGEJ-specific eQTL rs2240191 (red dot) was identified as top locus in epithelial cell-specific eQTLs (upper) and 7th locus in GWAS (lower) data set GCST90018841. The horizontal dotted lines represented P ≤ 0.05 (upper) and P ≤ 0.00001 (lower). The vertical dotted lines represented the same peak both in eQTL and GWAS data set. Genes from 113.1 Mb to 113.6 Mb on chromosome 12 were showed up in the box and susceptibility gene RASAL1 was highlighted in red.

**Supplementary Figure 7. Protein-coding prognosis markers except for by performing survival analysis.** **(A)** Kaplan-Meier survival curves for patients with different genotypes of eSNPs obtained which potentially regulated protein-coding gene expression. HR (95% CI) was computed with multivariate Cox hazard proportion model. Blue curves represented homozygote reference, red curves represented heterozygote and homozygote alteration.

**Supplementary Figure 8. Differential expression analysis of transcription factors and ACGEJ-specific eGenes.** **(A)** Volcano plot of differential expression analysis of transcription factors. Expression was adjusted by CIBERSORTx and filtered by quality control. Red, blue and grey dots represent up-regulated, down-regulated and not significantly regulated transcription factors, respectively. The X- and Y-axis represents expression fold change and adjusted *P* value, respectively. **(B)** Volcano plot of differential expression analysis of ACGEJ-specific eGenes. Expression was adjusted by CIBERSORTx and filtered by quality control. Red, blue and grey dots represent up-regulated, down-regulated and not significantly regulated eGenes, respectively. The X- and Y-axis represents expression fold change and adjusted *P* value, respectively.
